# Provincial legislative and regulatory standards for pain assessment and management in long-term care homes: a scoping review and in-depth case analysis

**DOI:** 10.1186/s12877-020-01758-7

**Published:** 2020-11-09

**Authors:** Natasha L. Gallant, Allie Peckham, Gregory Marchildon, Thomas Hadjistavropoulos, Blair Roblin, Rhonda J. N. Stopyn

**Affiliations:** 1 Department of Psychology and Center on Aging and Health, University of Regina, 3737 Wascana Parkway, Regina, Saskatchewan S4S 0A2 Canada; 2 Edson College of Nursing and Health Innovation, Arizona State University, 550 N 3rd Street, Phoenix, AZ 85004 USA; 3 Institute of Health Policy, Management and Evaluation, University of Toronto, 425–155 College Street, Toronto, Ontario M5T 3M6 Canada

**Keywords:** Canada, Long-term care, Pain assessment, Pain management, Quality standards

## Abstract

**Background:**

Among Canadian residents living in long-term care (LTC) facilities, and especially among those with limited ability to communicate due to dementia, pain remains underassessed and undermanaged. Although evidence-based clinical guidelines for the assessment and management of pain exist, these clinical guidelines are not widely implemented in LTC facilities. A relatively unexplored avenue for change is the influence that statutes and regulations could exert on pain practices within LTC. This review is therefore aimed at identifying the current landscape of policy levers used across Canada to assess and manage pain among LTC residents and to evaluate the extent to which they are concordant with evidence-based clinical guidelines proposed by an international consensus group consisting of both geriatric pain and public policy experts.

**Methods:**

Using scoping review methodology, a search for peer-reviewed journal articles and government documents pertaining to pain in Canadian LTC facilities was carried out. This scoping review was complemented by an in-depth case analysis of Alberta, Saskatchewan, and Ontario statutes and regulations.

**Results:**

Across provinces, pain was highly prevalent and was associated with adverse consequences among LTC residents. The considerable benefits of using a standardized pain assessment protocol, along with the barriers in implementing such a protocol, were identified. For most provinces, pain assessment and management in LTC residents was not specifically addressed in their statutes or regulations. In Alberta, Saskatchewan, and Ontario, regulations mandate the use of the interRAI suite of assessment tools for the assessment and reporting of pain.

**Conclusion:**

The prevalence of pain and the benefits of implementing standardized pain assessment protocols has been reported in the research literature. Despite occasional references to pain, however, existing regulations do not recommend assessments of pain at the frequency specified by experts. Insufficient direction on the use of specialized pain assessment tools (especially in the case of those with limited ability to communicate) that minimize reliance on subjective judgements was also identified in current regulations. Existing policies therefore fail to adequately address the underassessment and undermanagement of pain in older adults residing in LTC facilities in ways that are aligned with expert consensus.

**Supplementary Information:**

The online version contains supplementary material available at 10.1186/s12877-020-01758-7

## Background

### Pain assessment & management

The underassessment and undermanagement of pain in older adults, and especially those with dementia, residing in long-term care (LTC) facilities has been identified as an increasingly important issue [1]. First, pain is highly prevalent among older adults in LTC facilities [2]. In fact, pain has been documented as being more common than any other chronic condition in LTC [3]. Second, the underassessment of pain in this population is well-documented [4]. It has been demonstrated, for instance, that treating physicians were not able to detect pain in 34% of LTC residents known to be suffering from pain [5]. Importantly, inadequate pain assessments can result in unnecessary suffering and missed opportunities for early intervention among LTC residents [6, 7]. Third, considerable evidence suggests that seniors in LTC are undertreated for pain [8–11]. Unsurprisingly, the undermanagement of pain in LTC is most significant for persons with cognitive impairments and limited ability to communicate [12, 13]. The undermanagement of pain in is also of concern given that unmanaged pain in this population is associated with decreased socialization [14], exacerbated symptoms of dementia [10], worsened mood and depression [15, 16], as well as increased care needs and costs at the organizational level [10].

Adequate assessment and management of pain are therefore necessary if we are to improve health status, quality of life, and satisfaction of residents as well as their family members and care team [5, 17]. Researchers, practitioners, and decision makers who support a rapidly aging population recognize the importance of the underassessment and undermanagement of pain in LTC [18–20]. Internationally, several clinical guidelines have been developed for pain assessment and management in LTC [14, 21–29]. Observational assessment tools that are specialized, validated, and easy-to-use have been made available (e.g. [30],); practical approaches to assessment have been articulated (e.g., assess pain before and after an analgesic trial, consult with collaborative informants [31];); and minimum frequencies for pain assessment in LTC have been recommended [32]. Furthermore, many clinical guidelines on pain assessment in LTC emphasize the need to use specialized observational tools that have been developed for the assessment of pain [e.g., 22, 28].

Despite their development and availability, however, such approaches and protocols are not widely implemented in LTC practice. To gain a better understanding of this issue, a group of geriatric pain and public policy experts set out to examine the reasons behind the inadequate implementation of interventions to facilitate pain assessment and management in LTC [32]. They concluded that lack of satisfactory implementation of pre-existing guidelines was the result of excessive demands placed on and limited resources available to LTC staff. As a response to this finding, clinical guidelines comprising of minimum standards that were thought to be feasible and appropriate for managing pain in LTC were articulated [32]: (1) all LTC residents must be assessed for pain on admission and at least once a week thereafter with some residents requiring more frequent assessments; (2) a treatment plan must be documented within 24 h of pain problem identification with reassessment of outcomes and side effects within another 24 h; and (3) these assessments must involve a well-validated standardized assessment tool and, for residents unable to self-report pain, observational tools should be used.

To examine the desirability, validity, and feasibility of these guidelines, Hadjistavropoulos and colleagues surveyed administrators and front-line staff working in LTC across three Canadian provinces [32]. The results of this survey supported the proposed guidelines. That is, most participants reported that the guidelines were both highly desirable and feasible. Moreover, recent research suggests that the frequency of pain assessments can be successfully increased with the implementation of such systematic pain assessment practices in LTC facilities [33]. This increase in pain assessments was accomplished, for example, by dividing the task of pain assessments across front-line LTC staff to avoid an increase in workload among a select subgroup of staff members. It is important to find ways of improving the frequency of pain assessments in LTC environments as these improvements have been associated with reductions in polypharmacy and better pain management for residents as well as reduced job stress and burnout for staff [33, 34].

### Provincial Legislative & Regulatory Standards

Another reason for the lack of widespread implementation of standardized pain assessment and management protocols in LTC practice may be insufficient statutes and regulations that would facilitate their implementation. The use of public policy levers has previously been shown to be an essential component in achieving change within LTC settings. For example, the enactment of the 1987 Omnibus Budget Reconciliation Act in the United States resulted in a reduction in the use of unnecessary psychotropic medications in LTC facilities [35–37]. Thus, in a similar manner, increased attention needs to be placed on the public policy levers capable of facilitating the successful implementation of approaches and protocols to pain assessment and management within the Canadian context.

Regulatory instruments are among the most powerful tools at any government’s disposal [38]. Such regulatory instruments take on two very different forms: One is a law that must be passed by a legislature in the full glare of the public eye, including media scrutiny, and the other is a regulation that is enacted by the executive branch of government with very limited public scrutiny. A statute is a written law, enacted by a provincial legislature either commanding or prohibiting some action or declaring a policy. A regulation sets out the details of how the statute will be applied and enforced.

In Canada, the authority to prescribe regulations is often governed, first, by general statutory enactments and, second, by the enabling Act. For example, Ontario’s Interpretation Act states the general principle (s.23) that the “Lieutenant Governor in Council [i.e., cabinet] may make regulations for the due enforcement and carrying into effect of any Act of the Legislature” and the Regulations Act states (s.10) that “the Lieutenant Governor in Council may make regulations (b) prescribing the form, arrangement and scheme of regulations.”

The statutes that govern LTC in each province go further to specify the scope of the regulations under those enabling Acts. To illustrate, the Ontario Long-Term Care Homes Act provides (s.38) that the Lieutenant Governor in Council (i.e., the provincial cabinet) may make regulations governing matters as broad as the mission statements of LTC homes (subsection 2(d)) and as narrow as the temperature requirements in each home (subsection 2(b)). In other words, the cabinet can make regulations without reference to the more involved process of all-party reviews by committees of the legislatures and the scrutiny of formal votes through provincial legislative assemblies.

All provincial governments have introduced statutes concerning the licensure, funding, operation, care, and inspection requirements of LTC homes and have provided for the promulgation of regulations to govern these matters [39, 40]. Most Canadian jurisdictions (with the exception of Prince Edward Island and Quebec) use the interRAI continuing care reporting system to input demographic, clinical, functional, and resource utilization data on individuals receiving continuing care in hospitals and LTC facilities across Canada. In recent years, these data have been collected using the interRAI–Minimum Data Set 2.0 (RAI-MDS 2.0 (e.g. [41, 42],). The RAI-MDS is a clinical assessment tool developed by interRAI, a not-for-profit collaboration of clinicians, researchers, and health administrators from over 30 countries committed to improving services for vulnerable populations such as older persons [43]. In Canada, the Canadian Institute of Health Information (CIHI) is responsible for collecting these data and publicly disseminating summary reports.

### Purpose

This policy review was aimed at clarifying the Canadian statutes and regulations that support the appropriate assessment and management of pain in LTC and the extent to which these statutes and regulations are consistent with evidence-based clinical guidelines. To do so, a Canadian perspective on the way in which pain assessment and management approaches are embedded within quality standards in LTC facilities is provided with a more detailed focus on Alberta, Saskatchewan, and Ontario as provincial case studies.

## Methods

Prior to completing the scoping review, preliminary searches for all Canadian jurisdictions were conducted to identify the statutes and regulations specific to pain assessment and management that governed LTC. Due to existing formal agreements with the relevant provincial ministries of health as well as reasons of expertise and feasibility, an in-depth case analysis of Alberta, Saskatchewan, and Ontario’s statutes and regulations was conducted.

Given the exploratory nature of this study’s research question, a scoping review using the stepwise approach outlined by Arksey and O’Malley (2005) was completed [44, 45]. The purpose of this scoping review was to assess primary and secondary literature regarding legislative and regulatory standards related to assessing and managing pain among residents living in Canadian LTC facilities. To assess the primary literature, PubMed, Ovid, and ProQuest platforms were used to search databases such as PsycINFO, MEDLINE, EMBASE, ProQuest Dissertations & Theses Global, Sociological Abstracts, Worldwide Political Science Abstracts, and Education Resources Information Centre. To assess the secondary literature, Canadian government documents were retrieved using the Canadian Research Index as well as custom Google searches. For both types of searches, the following search string was used to search the titles, abstracts, and keywords of each database or search engine: legislat* AND regulat* AND manag* AND quality standard* AND pain AND assess* AND (residential long-term care OR nursing home* OR long-term care home* OR facility based long-term care).

Once duplicates were removed, two reviewers screened titles and abstracts to determine if documents met the following criteria: (1) specific to the Canadian context; (2) focused on pain in LTC; (3) available as a full-text peer-reviewed journal article or government document; (4) published on or after January 1, 2008; and (5) published in English. Each reviewer independently screened titles and abstracts of approximately half of the records identified and uncertainties about inclusion for records were resolved by consulting with the other reviewer to reach a consensus. Based on the screening of titles and abstracts, eligible full-text records were reviewed by the primary reviewer to determine inclusion in the review.

## Results

### Scoping review

**Fig. 1:**
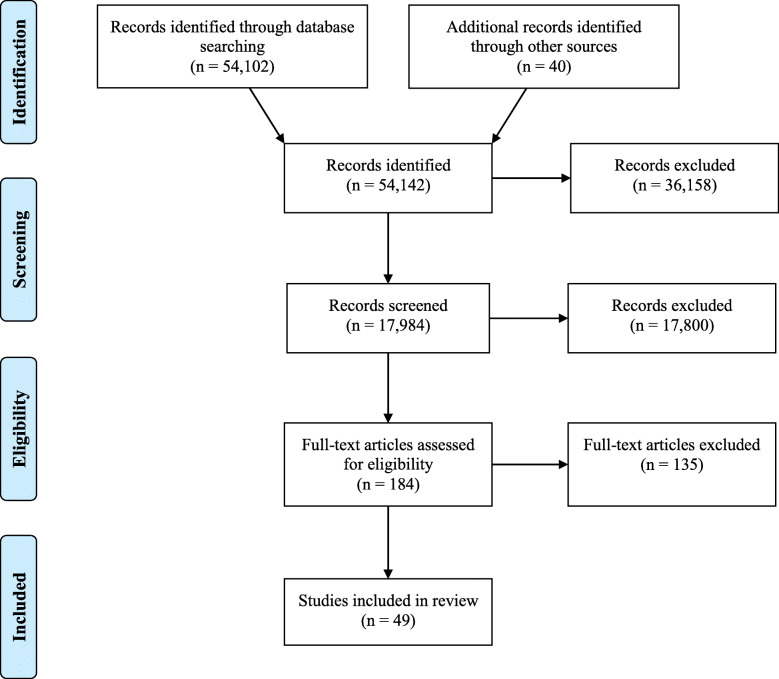
PRISMA diagram for scoping review

For the scoping review, the original search was completed in June 2018 and an updated search was completed in June 2020. The searches resulted in 54,102 peer-reviewed journal articles and 40 government documents. Once duplicates were removed, titles and abstracts were screened, and full-text articles were reviewed, a total of 49 records were retained for this review (see Fig. 1).

#### Primary literature

A summary of pain-related data extracted from each of these peer-reviewed journal articles is provided in Table 1. Pain prevalence rates within Canadian LTC facilities ranged from 27.1 to 75.6%, but it is important to note that methodological differences in definitions of pain, instruments used to measure pain, and populations examined existed [40, 51, 60, 64, 69]. Pain has also been shown to increase from admission to the end of residents’ stay in LTC [47]. Among Canadian LTC residents, inadequately managed pain has been associated with interference in daily functioning, the onset of delirium, and increased transfers to hospital [47, 59, 70].

**Table 1:** Overview of peer-reviewed journal articles identified for the scoping review

Reference	Jurisdiction	Brief Description of Methodology	Findings on Pain Assessment & Management in LTC
Bainbridge & Seow (2018) [46]	Ontario	Bereaved caregivers (*N* = 1153) completed the CaregiverVoice Survey	• Caregivers rated the relief of physical pain for residents as excellent (~ 52.5%), very good (~ 7.5%), good (~ 22.5%), fair (~ 12.5%), and poor (~ 5%)
Cheung et al. (2018) [47]	Ontario	LTC residents (*N* = 3897) were assessed using the RAI-MDS 2.0 at admission and repeated quarterly until death or discharge	• The proportion of residents reporting pain increased from baseline (42.3%) to follow-up (49.7%; *p* < .001) • The presence of pain was significantly associated with the onset of delirium (OR: 1.64, *p* < .001)
Estabrooks et al. (2013) [40]	Alberta, Saskatchewan, Manitoba	RAI-MDS 2.0 completed for LTC residents (*N* = 5196)	• 27.1% of LTC residents had daily pain • Alberta showed higher rates of worsening pain compared to Saskatchewan and Manitoba • Public facilities also had higher rates of worsening pain compared to private-for-profit and voluntary facilities
Fuchs-Lacelle et al. (2008) [34]	Saskatchewan	Nursing staff regularly assessed residents with dementia using the PACSLAC (*N* = 89) or using a control measure (*N* = 84)	• Regular assessments using the PACSLAC resulted in improved pain management practices and reduced observable pain behaviours • Nursing staff who regularly used the PACSLAC reported lower levels of stress and burnout
Gagnon et al. (2013) [48]	Saskatchewan	LTC staff (*N* = 148) evaluated a pain assessment training video and completed pain-related measures	• Pain assessment knowledge increased after staff viewed the training video • Staff thought positively about the content and quality of the training video • Barriers to implementing pain practices demonstrated in the training video included time, workload, and resistance to change
Ghandehari et al. (2013) [49]	Saskatchewan	LTC staff (*N* = 131) participated in an in-person pain education program or a control education program	• Pain knowledge, psychological beliefs about pain, and beliefs about pain and aging improved after staff participated in the education program • Barriers to implementing pain practices suggested in the education program included difficult patient characteristics, time, resources, and communication breakdown across professions
Hadjistavropoulos et al. (2010) [23]	Saskatchewan	Review of clinical guidelines for pain assessment among LTC residents	• Only 4.2% of Canadian physiotherapists were working in LTC settings • Physiotherapists can play a key role in assessing and managing pain among LTC residents
Hadjistavropoulos et al. (2011) [32]	British Columbia, Saskatchewan, Ontario	Stakeholders (*N* = 168) completed a Stakeholder Feedback Questionnaire regarding the possibility of implementing pain-related guidelines	• Stakeholders from Saskatchewan believed the policy recommendation of implementing a multidisciplinary geriatrics team less feasible compared to stakeholders from British Columbia and Ontario • Care aides rated the clinical recommendation of a documented treatment plan for residents with moderate-to-severe pain as more helpful compared to nurses • Care aides rated the policy recommendation of implementing a multidisciplinary geriatrics team and of reporting on results using a consistent set of performance measures as more feasible than did nurses and administrators
Hadjistavropoulos et al. (2014) [30]	Saskatchewan	Medication administration in LTC residents (*N* = 64) as well as interviews and focus groups with LTC staff (*N* = 19)	• LTC residents who were a part of a pain assessment protocol were given fewer psychotropic medications than residents in the control group • Protocol implementation resulted in more careful pain evaluation of LTC residents, better communication with physicians, and greater appropriate prescribing of medications
Hadjistavropoulos et al. (2016) [33]	Canada	Two LTC facilities evaluated by quality indicators for pain as well as interviews and focus groups with LTC staff (*N* = 34)	• Pain was assessed more frequently following implementation of pain assessment program • Staff reported a positive impact following pain assessment program implementation and maintenance • Implementation success was dependent on management support and staff willingness • No changes in percentages of patients with moderate-to-severe pain
Helmer-Smith et al. (2020) [50]	Ontario	eConsult cases (*N* = 24) by primary care providers in LTC and focus groups (*N* = 4) on eConsult use in LTC homes	• Specialists (including Pain Medicine) reported benefits and feasibility of using the eConsult service
Hill et al. (2019) [71]	Alberta, Saskatchewan, Manitoba, Ontario, Quebec	Current national and provincial palliative care guiding documents (*N* = 25)	• Ineffective pain management was one of the clinical issues that stimulated the development of new guiding documents for palliative care • The physical domain—which centers around pain and symptom management, maintaining cognition, and physical care—was addressed by 56% of guiding documents
Hirdes et al. (2011) [51]	Yukon, British Columbia, Saskatchewan, Manitoba, Ontario, Nova Scotia, Newfoundland & Labrador	Census data available from Statistics Canada’s Canadian Socioeconomic Information Management system regarding LTC residents (*N* = 128,168)	• Pain was commonly reported among LTC residents, such as 54.1 and 4.9% of residents in Saskatchewan who reported mild-to-moderate and excruciating pain, respectively
Hunter et al. (2020) [52]	Alberta, Saskatchewan, Manitoba, Ontario	Professional and non-professional LTC staff (*N* = 228) completed a survey assessing qualities regarding palliative care	• LTC staff are likely ready to embrace a palliative care mandate as indicated by their emotional well-being
Kaasalainen et al. (2010) [53]	Ontario	Interviews or focus groups were conducted with and survey was completed by pharmacists (*N* = 2), and physiotherapists (*N* = 2), administrators (N = 4), physicians (*N* = 4), care aides (*N* = 20), and nurses (*N* = 21)	• Based on the survey, barriers to pain assessment included residents’ limited ability to self-report their pain, residents’ reluctance to report pain, and inadequate time for staff • Based on interviews and focus groups, barriers were organized at the resident/family (e.g., residents’ inability to communicate), healthcare provider (e.g., staff not believing residents’ report of pain), and system (e.g., time constraints) levels
Kaasalainen et al. (2016) [54]	Canada	LTC residents (*N* = 345) participated in a pain management team led by a nurse practitioner, were led by a nurse practitioner without a pain management team, or had no nurse practitioner or pain management team	• Residents receiving care from the pain management team led by a nurse practitioner experienced reduced pain and improved functional status • Clinical practice behaviours improved in the nurse-led pain management team • Barriers to effective team functioning included lack of staff knowledge about medication management, establishing the role of the nurse practitioner on the team, and effectively communicating about residents’ pain across staff
Lane et al. (2019) [55]	Ontario	Database review of LTC residents (*N* = 12,334) receiving disability assessments at admission and subsequent 2 years	• Daily pain was not associated with greater disability at admission or over time
Mashouri et al. (2020) [56]	Ontario	LTC homes (*N* = 594) classified based on RAI-MDS quality indicators to predict performance	• Quality indicator of worsening pain predicted the LTC homes needing improvement
McArthur et al. (2019) [57]	Alberta, Ontario, British Columbia, Manitoba, Nova Scotia, Newfoundland, Saskatchewan, Yukon	RAI-MDS 2.0 data on Complex Continuing Care (*N* = 2455) and LTC residents (*N* = 142,386) who are comatose	• Lower proportion of residents who are comatose had documented pain compared to residents who are not comatose
McCleary et al. (2018) [58]	Alberta, Saskatchewan, Manitoba, Ontario	Focus groups with LTC staff (*N* = 77) and family members of persons with dementia living in LTC (*N* = 19)	• Staff and families thought that end-of-life pain management was critically important for residents • Staff reported difficulties in assessing pain among residents with dementia • Staff and families believed that pain assessment and management for residents with or without dementia were more accurate when they knew the resident well
Nemiroff et al. (2019) [59]	Nova Scotia	Review of LTC resident charts and database notes (*N* = 748) from time periods	• Decisions to transfer LTC residents to hospital were influenced by inadequate pain control at the LTC as well as requests by family members or residents, inability to contact physician, injury, management and symptom control, and palliation
Ramage-Morin (2008) [60]	Canada	Data from LTC residents (*N* = 2287) were obtained from the data from the National Population Health Survey	• 37.9% of LTC residents reported experiencing chronic pain, with women experiencing higher rates of chronic pain than men • Of LTC residents reporting chronic pain, reported pain intensities were mild for 22.4%, moderate for 50.0%, and severe for 27.6% of residents • Pain was found to interfere more with daily activities for those LTC residents who reported moderate or severe pain compared to those who reported mild pain
Rosenberg et al. (2019) [61]	British Columbia	Individuals (*N* = 380) receiving home-based primary geriatric care in the community assessed using frailty and quality of life measures	• Chronic pain did not predict transfer to LTC facilities
Senderovich et al. (2019) [62]	International	Literature search on efficacy of herpes zoster vaccine in LTC from 2013 to 2018 (*N* = 10)	• Vaccine was associated with shorter pain duration and reductions in pain severity
Tadrous et al. (2020) [63]	Ontario	LTC residents (*N* = 5363) who were provided with antipsychotics using academic detailing vs. usual care	• Residents who received academic detailing reported a reduction in pain compared to those who received usual care
Turcotte et al. (2018) [64]	Ontario	LTC residents with Alzheimer’s disease or related dementias (*N* = 107,381) who were assessed using the RAI-MDS 2.0	• Of LTC residents with Alzheimer’s disease and related dementias, 57.9% reported no pain, 39.3% reported mild to moderate pain, and 2.8% reported excruciating pain
Watt-Watson et al. (2013) [21]	Canada	Documents were evaluated for entry-to-practice competency requirements related to pain knowledge, skill, or judgment (*N* = 21)	• Pain-specific competencies were only listed for dentistry (*N* = 2), nursing (*N* = 9), and veterinary (*N* = 9) documents
Wilchesky et al. (2018) [65]	Quebec	Medication intervention among residents with severe dementia (*N* = 44)	• Slight increase in pain from pre-intervention (8.1) to post follow up (9.7, *p* < .0001) but unclear if increase is the result of intervention or disease progression
Wilkinson et al. (2019) [66]	Ontario	RAI-MDS 2.0 completed for LTC residents (*N* = 614)	• Improvement in quality performance (including pain management) over time in LTC homes from 2012 to 2017
Yoon et al. (2018) [67]	Alberta, Manitoba, Ontario, New Brunswick	LTC residents (*N* = 559) received a standardized oral health examination	• Less than 20% of residents with dentures reported pain in teeth or jaw pain but self-report may underestimate prevalence
Yoon et al. (2020) [68]	Alberta	Nine focus groups with LTC staff (*N* = 44) on residents’ oral and dental health	• Staff primarily relied on resident self-reports of mouth pain • Pain was inferred from changes in eating and non-verbal expressions of pain (i.e., facial grimacing)
Yu et al. (2020) [69]	Ontario	Newly admitted LTC residents (*N* = 4853) following a stroke assessed for care needs and mortality	• Female LTC residents more likely to have pain compared to male LTC residents

Regular assessments using the PACSLAC, or other specialized pain assessment tools, can result in reduced pain, more frequent pain assessments, and improved pain management practices such as more appropriate prescribing of medications [6, 33, 34, 54]. Furthermore, nursing staff who regularly assess for pain using the PACSLAC reported lower stress and burnout levels as well as better communication with physicians [6, 34]. However, as previously mentioned, barriers in implementing standardized pain assessment protocols in LTC have been identified: time constraints, heavy workload, lack of resources, resistance to change by nursing staff, lack of management support, communication breakdown across professions, and communication difficulties between residents and nursing staff regarding pain [33, 48, 49, 53].

Solutions in addressing these barriers have also been proposed. For example, pain assessment knowledge increases and, consequently, pain practices improve among nursing staff following in-person or video-based pain assessment training programs [48, 49]. Other suggestions for improving pain assessment and management practices in LTC include an increase of physiotherapists embedded within LTC facilities and implementing a virtual consultation service to communicate with specialist physicians working in pain medicine [23, 50]. Finally, ineffective pain assessment and management practices has influenced the development of clinical guidelines in palliative care [52, 71] given the importance placed on appropriate end-of-life management of pain among residents in LTC facilities [60].

#### Secondary literature

Findings from the secondary literature included in this scoping review is summarized in Additional file 1: Appendix A. For each province, the relevant statutes and regulations along with a brief description of the organization and administration of LTC homes within the province are listed. The basic provisions related to resident care and any specific references to pain are also identified. For example, Newfoundland and Labrador has issued operational standards [72], which state that, (i) with respect to a patient’s integrated care plan, performance measures are to include “baseline pain assessment” (Standard 1.2(9)) and, (ii) with respect to care of a dying resident, “pain assessment, management and relief are provided as required” (Standard 13.4). Similarly, New Brunswick has issued a standards manual [73] that requires LTC facilities (i) to continually assess, plan, design, and implement programs, and specifically includes a pain management program, and (ii) to provide in-service training on pain management for all employees. Nova Scotia’s program requirements [74] specify that “residents have an interdisciplinary assessment of […] pain and discomfort” within the first 2 weeks of admission and that “resident care protocols, based on current leading practices, are developed, implemented, monitored and regularly evaluated with particular emphasis on […] pain.” In most provinces, however, the word “pain” is not specifically mentioned in their regulations (e.g., British Columbia [75], Manitoba [76], Prince Edward Island [77]), although issues related to pain may be recognized and addressed in the context of the care assessment or plan that is typically the subject of regulatory provisions.

Nonetheless, measurement and quality indicators (e.g., RAI-MDS) are in place across Canada for assessing residents who may have pain [78, 79]. No standardized best practices for pain assessment and management, besides the administration of the RAI-MDS as indicated above, however, exists in any Canadian jurisdiction. When using the RAI-MDS for the assessment of pain, clinicians are instructed to ask simple and direct questions about the experience of pain and to rely on self-report when possible. Other sources of information can include clinician observations or caregivers.

With regards to inspections, deterrence- or compliance-based approaches are used across jurisdictions. As an example, Ontario has been described as using a deterrence-based approach with prescribed and unannounced inspections [80]. Ontario also has public reporting of certain inspection results and offers a complex system of sanctions—fines, withholding funds, appointing temporary managers, and revoking licenses. Deterrence-based approaches to inspections are more effective as compliance-based approaches do not influence front-line staff making decisions that directly affect quality standards [81].

### In-depth case study of Alberta, Saskatchewan, and Ontario

The in-depth case studies provided a more detailed examination of practice guidelines, protocols, and other authoritative standards required of practitioners involved in pain assessment and management in LTC facilities. Table 2 summarizes the results of the in-depth case analysis of Alberta, Saskatchewan, and Ontario as it pertains to pain assessment and management in LTC. As well, Table 2 includes pain-related interRAI indicators as reported by CIHI.

**Table 2:** References to regulatory instruments and associated outcomes for pain in Alberta, Saskatchewan, and Ontario

Province	Specific regulatory instrument for pain	interRAI instrument mandated for pain	Experiencing Pain in Long-Term Care (2018–2019)^**a**^	Experiencing Worsened Pain in Long-Term Care (2018–2019)^**a**^	Other instruments mandated that are not specific to pain
Alberta	No	Yes	7.4%	13.5%	Long-Term Care Accommodation Standards and Checklist, 2010 Continuing Care Health Service Standards, 2016
Saskatchewan	No	Yes	9.0%	11.8%	Program Guidelines for Special-care Homes, 2016
Ontario	O. Reg. 79/10	Yes	5.2%	9.7%	N/A

^a^ Data obtained using CIHI’s Your Health System interactive tool that provides recent interRAI indicator results [39]

#### Alberta

In Alberta, the Ministry of Health is responsible for setting strategic direction; establishing legislation, policies, and provincial standards of health care; and measuring and reporting on quality and performance across the health system. LTC facilities are governed under the Nursing Homes Act, 2000. In addition to governing the terms of contracts, the Act lays out general terms for the operation of LTC homes. The Nursing Home General Regulation under the Act defines basic care (section 2) as a wide variety of ancillary accommodation, meals, and personal services but does not mention pain assessment or management procedures. The Nursing Home Operation Regulation delineates the admission policies of nursing homes, resident assessments, staffing requirements and qualifications.

Alberta Health Services, directly accountable to the Minister of Health and Alberta Health (the Department of Health), is responsible for the delivery of health care in the province and for establishing operational policy. Subsection 4(2) of the Nursing Home General Regulation requires that LTC facility operators comply with two sets of standards set by the Alberta Health Services. The first is the Long-Term Care Accommodation Standards and Checklist, 2010 [41], which is set by Alberta Health’s Standards Compliance and Licensing Branch and deals primarily with the physical standards around nursing homes. The second is the Continuing Care Health Service Standards [CCHSS], 2016 [42], set by Alberta Health’s Continuing Care Branch. The CCHSS stipulate the minimum requirement that operators in the continuing care system must meet.

Within the residential care sector, the CCHSS apply to both LTC homes and publicly funded supportive living facilities, and pertain more to person-centered care planning, assessment, and case management. Pain management was mentioned under the operational processes in Standard 1.21 B (i.e., “Operational policies and processes shall include pain assessment and management”) and is implemented and operationalized at the LTC facility level [42]. Subsection 1.0 (1.1) of the CCHSS mandates the use of the interRAI instruments by all operators (defined as those receiving public funding for the provision of health care) that are subject to the Nursing Home General Regulation. By incorporating the interRAI instruments, operators of LTC facilities must comply with all the requirements related to pain assessment and management contained therein.

#### Saskatchewan

In Saskatchewan, the overriding legislation for LTC facilities is the Provincial Health Authority Act which came into force in December 2017. The Housing and Special-care Homes Regulations govern the administrative procedures under the Act, including nursing care (section 4), medications (section 8) and food services (section 11). The Facilities Designation Regulations under the Act describe the services to be provided by various health-related facilities in the province and section 12 states that a facility designated as a special-care home must provide personal care or nursing care to individuals who reside in the facility. Standard 1.4(u) of the guideline states “every effort is made to recognize, assess and appropriately manage pain”. However, pain management protocol is left up to the discretion of the individual LTC facilities [82].

The provincial Ministry of Health publishes a manual entitled, *Program Guidelines for Special-care Homes [Guidelines]* [83], that was last updated in May 2016. Pursuant to the Facility Designation Regulations, section 17(2), all special-care homes are required to operate in accordance with the standards set out in the *Guidelines*. The quality indicators include assessing worsened pain. However, the Provincial Auditor’s report noted that the measures tracked did not provide insight into the care practices (including pain management) of special-care homes [84].

The *Guidelines* provide detail regarding access to service; types of care to be provided; assessment procedures; requirements and qualifications of nursing and personal care providers; resident care plans; support services; and nutrition services. No specific references to pain in the statute or regulations were identified. However, the *Guidelines* incorporate (by virtue of Section 9a.1 Resident Assessment Tool) the interRAI suite, specifically RAI MDS 2.0, to all resident assessments.

#### Ontario

Ontario appears to be the only province where pain assessment or management directives are inserted directly into its regulations. The Long-Term Care Homes Act, 2007 is the governing statute in Ontario, furthered by General Regulation, O.R. 79/10. Ontario’s General Regulation 79/10 refers to pain in terms of the plan of care (s.26), required programs (s.48), pain management (s.52), and training of direct care staff (s.221). The Ministry of Health and Long-Term Care is responsible for licensing, inspecting, and setting the fees for LTC homes [80]. Homes can be owned by private corporations, non-profit organizations, or municipal governments. In terms of the provision of care, the Act dictates that a plan of care be devised for each resident and prescribes the care services to be available in all LTC homes. The General Regulation provides further detail as to the requirements of the care plan and all nursing and support services.

The General Regulation also makes specific reference to pain in several sections. With respect to the requirement for a plan of care for each resident, the regulation states (s.26) that the plan involve an interdisciplinary assessment of “health conditions, including allergies, pain, risk of falls and other special needs.” Under required programs (s. 48), it stipulates that every home licensee have an interdisciplinary program in place that includes “a pain management program to identify pain in residents and manage pain.” The specifics of these programs appear to be left to the responsibility of each LTC facility [80]. Finally, section 221 of the General Regulation requires that training of all staff who provide direct care to residents must include “pain management, including recognition of specific and non-specific signs of pain.”

Ontario’s LTC homes are subject to the requirements of RAI-MDS 2.0, originally initiated in June 2005 [85]. RAI-MDS 2.0 has become the assessment tool for admission, quarterly and annual assessments, and significant changes in health status for each resident. According to the Ministry of Health and Long-Term Care’s Guide to the Long-Term Care Homes Act [85], the interRAI suite derives its authority in Ontario from section 26(3) of the General Regulation, which specifies the domains of care that must be included in the assessment and is the basis of the plan of care.

## Discussion

Several professional organizations are working to identify best practices and guidelines to support quality of pain assessment for LTC residents [40]. The Registered Nurses’ Association of Ontario (2013), for example, released a report outlining best-practice guidelines for the assessment and management of pain [86]. Similarly, the Ontario Association of Non-Profit Homes and Services (2010) detailed a pain management approach [87]. Furthermore, accreditation standards for LTC facilities in Canada consider pain, but they have not succeeded in providing specific guidance about the use of cutting-edge assessment tools, especially for residents with limited ability to communicate. Despite these efforts, pain remains an unrecognized and undertreated condition in LTC settings [70, 88]. As such, leaving the specifics of quality improvement initiatives up to individual LTC facilities may be insufficient in addressing residents’ pain. Of note, documented evidence suggests that LTC facilities face numerous barriers when attempting to successfully implement quality improvement initiatives [32, 33]. It is for this reason that this study was aimed at exploring another avenue in facilitating quality improvement initiatives. That is, the role of Canadian statutes and regulations in supporting pain assessment and management in LTC was examined through a pan-Canadian scoping review as well as an in-depth case analysis of Alberta, Saskatchewan, and Ontario.

Based on the illustrated example of Ontario’s success in regulating LTC facilities, provincial government can use regulation as a vehicle to implement appropriate guidelines in LTCs. Specifically, provincial governments can proscribe specific standards and practices relative to pain assessment and management for LTC residents (e.g., increased frequency of pain assessments to match expert consensus guidelines [32]). Indeed, an examination of Ontario Regulation 79/10 under Ontario’s Long-Term Care Homes Act illustrates the detail with which regulations of health standards can be extended, including the frequency of bathing (s.33), denture cleaning (s.34), and toenail cutting (s.35). As such, regulations in all provinces could require, on a compulsory basis, the three evidence-based pain assessment requirements set out in this policy review [32]. The use of regulations to implement these guidelines would be a far less onerous process than legislative changes and, furthermore, be more suited to defining the application of guidelines in adequate detail. In addition, while some cost to operators of LTC facilities in implementing these changes will be incurred, they are likely to be minimal as illustrated in prior implementation research [33].

The most important finding of this study is that, in the provinces of Alberta, Saskatchewan, and Ontario, interRAI is referenced as the legal standard for pain assessment and management as mandated by LTC regulations in each province. According to the interRAI website, all provinces except Quebec, New Brunswick, and Prince Edward Island have mandated the RAI-MDS 2.0 [89]. According to the Government of New Brunswick’s website, New Brunswick also announced the adoption of the RAI assessment tool for use in nursing homes as of June 2015 [73]. The current mandated version for use in LTC facilities is the RAI-MDS 2.0. The Canadian Version of the RAI-MDS 2.0 User’s Manual [90] addresses the issue of pain assessment and management. That is, the RAI-MDS 2.0 includes an item to document pain frequency as “no pain,” “pain present but not in the past 3 days,” “present on 1 to 2 of the past 3 days,” and “present daily in the past 3 days.” Current pain intensity is documented as none, mild, moderate, severe, and unbearable. The pain frequency and intensity items can be summed to determine the Pain Scale score [91]. 

Although mandated by LTC regulations in most Canadian provinces, the RAI-MDS 2.0 still does not meet the recommended practice guidelines for pain assessment and management outlined at the beginning of this paper [32]. Of note, pain assessment is not the specific focus of the RAI-MDS or the suite of assessment tools [92–94]. The RAI-MDS 2.0 also does not require use of a standardized self-report tool such as a numeric rating scale or verbal-rating scale even though the use of clinical judgement in the absence of systematic specialized observation tools has been shown to result in pain being underdiagnosed [4–6, 12, 34]. Canada may be moving in that direction, however, as it is in the process of transitioning to the use of the interRAI Long-Term Care Facilities (LTCF) tool. The interRAI LTCF is an improvement from the RAI MDS 2.0 because it adds more information to the assessment process but still does not offer sufficient guidance for the optimization of pain assessments [95].

The use of the RAI-MDS may also lead to over- or under-reported pain [79, 80, 85, 96]. For instance, facility characteristics have been shown to be more important than resident characteristics in predicting agreement between RAI-MDS pain indicators and a “gold standard” examination by nurse raters [97]. Moreover, Proctor and Hirdes (2001) showed that the RAI-MDS suggested a lower prevalence of identified pain among LTC residents with higher levels of cognitive impairment despite the absence of differences in the prevalence of pain-related conditions [98]. Importantly, current regulations in most Canadian provinces specify that pain assessment should be conducted at minimum once every 3 months using the RAI-MDS [41, 42]. From a clinical standpoint, however, this frequency of pain assessment is insufficient as it has been recommended that pain should be assessed at minimum once a week using specialized tools [99]. Even the more recent interRAI LTCF does not provide guidance on the frequency of pain assessments. Finally, residents who had limited ability to communicate—due to, for example, dementia—have been routinely excluded from studies examining the reliability and validity of the interRAI Pain Scale [39, 91, 100]. This is especially problematic given that over 25% of nursing home residents are unable to self-report pain and to comprehend questions related to pain assessment [1].

There is little question concerning the importance of maintaining a minimum data set for quality assurance purposes within the context of LTC. In Canada, the maintenance of a minimum data set has mostly been accomplished through the use of the RAI-MDS. Based on the limitations mentioned above, the quality of RAI-MDS pain assessments would, however, be enhanced through the use of specialized pain assessment tools (e.g., the Pain Assessment Checklist for Seniors with Limited Ability to Communicate-II (PACSLAC-II [101, 102]), the Pain Assessment in Advanced Dementia (PAINAD [103]), the Abbey Pain Scale [104] and others as recommended by pre-existing guidelines). In fact, the national Nursing Home Pain Collaborative in the United States has expressed the opinion that specialized pain assessment tools, such as the PACSLAC, would be especially useful for facilitating completion of the RAI-MDS [22, 99]. In their consensus article, Hadjistavropoulos and colleagues suggested that routine screening assessments involving standardized tools would typically require less than 5 min to complete [32, 105]. This screening, of course, would be completed in addition to more comprehensive pain assessments undertaken at the time of admission or in other situations where it is clinically indicated. Once implemented, facilities could track pain levels over time and use these data as quality indicators to inform their quality improvement programs [32]. Therefore, one way of inciting further improvements in pain assessment and management might be regulatory changes mandating more frequent use of the RAI-MDS 2.0 in combination with one or more easy-to-administer standardized pain assessment tools [99].

Based on the findings of this study, future directions for research are proposed. First, the relationship between improved regulations and pain outcomes in the Canadian provincial context is warranted. Although this study’s findings are suggestive of the need for regulatory changes to improve the assessment and management of pain in LTC, this relationship needs to be systematically investigated. Second, future research should examine the usefulness of mandating training for front-line LTC staff in the assessment and management of pain. This training, which can contribute to the enhancement of cutting-edge pain assessment and management knowledge, could be provided not only via traditional workshops but also through other media, such as video-based or online formats [48, 49]. Such education is often necessary given that knowledge gaps among nursing staff are well-documented [106] and the specialized assessment of pain needed for residents with dementia is not yet provided in typical professional training programs for health professionals in Canada [21]. Third, our search strategy may have resulted in unidentified documents that could have informed the current scoping review. Future studies could involve either an environmental scan so as not to miss any relevant documents or a systematic review that would allow for a more comprehensive approach in identifying relevant documents. Furthermore, the exclusion of documents published in languages other than English may have resulted in limited input from the province of Québec, for which French is the official language, in this pan-Canadian review. Future studies should include, at minimum, documents in both English and French as these languages comprise Canada’s official languages. Finally, research aimed at comparing the approach of Canadian jurisdictions to other international jurisdictions would be a valuable contribution to our overall understanding of pain assessment and management in LTC settings.

## Conclusions

Policy interventions aimed at improving the quality of care can take a variety of forms, including regulatory requirements, carefully calibrated subsidies or other incentives to encourage certain behaviours, or accreditation requirements. From a policy perspective, constrained health budgets in the public sector put a premium on reducing the downstream cost of faulty diagnosis and treatment based on inadequate assessment and undermanagement of pain, a major problem among LTC residents with dementia. The policy remedy for this ailment is for governments to directly regulate reporting requirements for pain assessment and management. Addressing pain in LTC is an important avenue for policy work as significant discrepancies between current practices and internationally developed evidence-based clinical guidelines are noted. Improving the assessment and management of pain in LTC also fits within the broader goal of improving facility-based nursing care as well as the daily functioning and quality of life of LTC residents.

## Supplementary Information

**Additional file 1: Appendix A.** 10-province scan of LTC statutes and regulations. This additional file includes an appendix describing our results from a scan of long-term care statutes and regulations across the 10 Canadian provinces.

## Data Availability

Only publicly available documents were analyzed for this research. No other data was used.

## Abbreviations

CCHSS: Continuing Care Health Service Standards
CAPs: Clinical Assessment Protocols
Guidelines: Program Guidelines for Special-care Homes
LHINs: Local Health Integration Networks
LTC: Long-term care
L-SAA: Long-Term Care Home Service Accountability Agreement
PACSLAC-II: Pain Assessment Checklist for Seniors with Limited Ability to Communicate-II
PAINAD: Pain Assessment in Advanced Dementia
RAI-MDS: Resident Assessment Instrument-Minimum Data Set
