# Supplementary material for: Provincial legislative and regulatory standards for pain assessment and management in long-term care homes: a scoping review and in-depth case analysis
Source: BMC Geriatr. 2020 Nov 9;20:458. doi: 10.1186/s12877-020-01758-7 (PMC7650170; doi:10.1186/s12877-020-01758-7)
Supplement: Supplementary file 1 — Additional file 1: Appendix A. 10-province scan of LTC statutes and regulations. This additional file includes an appendix describing our results from a scan of long-term care statutes and regulations across the 10 Canadian provinces. [file 12877_2020_1758_MOESM1_ESM.docx]

*Appendix A.* 10-province scan of LTC statutes and regulations

**Newfoundland and Labrador**

Statutes:

Health Care Association Act, RSNL 1990, Chapter H-8

Regulations:

None specified.

Organization and Administration:

Nursing homes in Newfoundland & Labrador are residential long-term care facilities that provide accommodation, supervisory care, personal care and nursing and medical services on a 24-hour basis. Care and accommodation for these residents is subsidized by the Department of Health and Community Services. Assessment, waiting lists and admission into a nursing home are managed by the Regional Health Authorities (RHAs).

In 2005, the Provincial government issued Operational Standards for Long Term Care Facilities. As per the standards, nursing homes “provide residential care and accommodations to residents who have high care needs and require on-site professional nursing services.” Regional Health Authorities in the province are responsible for monitoring nursing homes to ensure compliance with the standards. The Department of Health and Community Services subsidizes nursing home care costs. The majority of nursing homes in Newfoundland and Labrador are public homes.

Provision of Care

The Operational Standards delineate the care services to be provided in LTC homes (section 3), as well as card access and delivery (section 4) and resident support services (section 6).

References to Pain Management, Education in governing or other instruments:

There is no specific reference to pain or pain management in the legislation. However, in 2005, the Provincial government issued Operational Standards for Long Term Care Facilities. These contain the following references to pain management:

- with respect to palliative care, there is a requirement that “the resident receives interventions to promote optimal comfort including the appropriate assessment and management of pain” (Standard 14.6).

- with respect to the patient’s integrated care plan, performance measures are to include “baseline pain assessment” (Standard 1.2(9)).

- with respect to care of the dying resident, performance measures are to include “pain assessment, management and relief are provided as required” (Standard 13.4).

**Nova Scotia**

Statutes:

Homes for Special Care Act, R.S., 1989, c.203, s.1.

Regulations:

Homes for Special Care Regulations, N.S. Reg. 127/77

Organization and Administration:

The Homes for Special Care Act governs the licensure and administration of “homes for special care,” including nursing homes. The regulations under the Act define homes for special care to include “a nursing home, a home for the aged, a home for the disabled and a residential care facility.” The Department of Health and Wellness, Continuing Care Division has created policies and monitoring standards to ensure consistent quality of care for services in LTC and in the community (www.novascotia.ca/dhw/ccs/policies-standards.asp).   As per the Department of Health and Wellness’s Long-term Care Policy Manual (Long-term Care Program Requirements: Nursing Homes and Residential Care Facilities), nursing homes “provide personal and/or skilled nursing care in a residential setting to individuals who require the availability of a registered nurse on-site at all times.” Nursing homes in Nova Scotia can be either publicly or privately owned and operated. Homes that receive public funding are required to have a license. Homes that do not receive public funding are not required to have a license.

Provision of Care

Responsibilities of homes for special care are delineated in the Long-term Care Policy Manual.

References to Pain Management, Education in governing or other instruments:

There are no specific references to pain in the legislation or regulations. However, Nova Scotia Health and Wellness has published the Long-Term Care Policy Manual in March 2016. This document details the requirements of resident assessments and individual plans of care but does not specifically mention pain.

**Prince Edward Island**

Statutes:

Community Care Facilities and Nursing Homes Act, RSPEI 1988, c C-13

Regulations:

General Regulations, PEI Reg EC391/84

Organization and Administration:

In P.E.I., LTC facilities include both publicly supported facilities, referred to as “manors,” and privately-owned facilities, which must be and licensed. The privately-owned facilities are referred to in the Community Care Facilities and Nursing Homes Act as “nursing homes” and defined as those homes that provide their services “for compensation”. Assessment and admission for nursing homes in P.E.I. are managed by the Department of Health. Privately owned nursing homes are governed by the Continuing Care and Nursing Home Act. They must hold a license from the Community Care Facilities and Nursing Homes Board, who also inspects these facilities on a regular basis. Manors experience a different regulation process. Manors must be accredited by Accreditation Canada, but are not subject to the same inspection regime as nursing homes.

Provision of Care

Subsection 27(a) of the General Regulation requires each resident to have an appraisal of medical, mental-emotional and behavioural status and to have a care plan formulated accordingly. Subsection (b) stipulates that the care services provided for the resident are appropriate to his needs as determined in the appraisal and care plan.

References to Pain Management, Education in governing or other instruments:

Pain is not specifically mentioned in the Regulation or the Act.

**New Brunswick**

Statutes:

Nursing Homes Act, SNB 2014, c 125

Regulations:

General Regulation, NB Reg 85-187

Contribution Regulation, NB Reg 2009-75

Services Regulation, NB Reg 2001-59

Organization and Administration:

The Nursing Homes Act in New Brunswick defines nursing homes as residential facilities operated for the purpose of supervisory, personal or nursing care for people who by reason of infirmity or mental or physical disability are not fully able to care for themselves. The Minister of Social Development is responsible for administration of the Act and is to appoint a Director of Nursing Homes Services. The Act provides for licensure of homes and defines the basic duties of the home operator. It also provides for government funding of homes, the inspection process and enforcement.

Provision of Care

The General Regulation under the Act, Part III, defines the Care Services, including medical, dental, dietary, activation and rehabilitation.

References to Pain Management, Education in governing or other instruments:

Section 18 (d) of the General Regulation provides that “a comprehensive care plan is developed for each resident upon admission, reviewed at least annually and evaluated on an ongoing basis.” There is no specific reference to pain or pain management.

In November 2016, the Department of Social Development issued a “Standards Manual, Nursing Home Services”, which contains the following provisions:

- the nursing home must continually assess, plan, design and implement programs and services to meet the current and future needs of the residents in order to achieve the best possible outcome, where such programs must include a Pain Management Program

- in-service training must be provided for all employees, which includes pain management.

**Quebec**

Statutes:

Act Respecting Health Services and Social Services, CQLR, Chapter S-4.2

Regulations:

Numerous regulations are found under the Act, though none appears to deal with pain management.

Organization and Administration:

Residential and Long-Term Care Centres are governed by the Act Respecting Health Services and Social Services. Nursing homes, or centres d’hebergement et de soins de longe duree (CHSLD), as they are called in Quebec, are residential facilities that provide 24-hour professional nursing care and supervision in a protective, supportive environment for people who have complex care needs and can no longer be cared for in their own homes. Admissions to subsidized nursing homes or beds (subsidized beds within a private nursing home) are managed by Local Community Service Centres in Quebec.

References to Pain Management, Education in governing or other instruments:

There does not appear to be any reference specifically to pain in the legislation or regulations.

**Ontario**

Statutes:

[Long-Term Care Homes Act, 2007, S.O. 2007, c. 8](https://www.ontario.ca/laws/statute/07l08)

Regulations:

General Regulation, O.R. 79/10

Organization and Administration:

In Ontario, the Ministry of Health and Long-Term Care is responsible for licensing, inspecting, and setting the fees for LTC homes. Homes are subject to the Long-Term Care Homes Act and General Regulation 79/10. Homes can be owned by private corporations, non-profit organizations, or municipal governments.

Provision of Care

The Act dictates that a plan of care be devised for each resident and prescribes the care services to be available in all LTC homes. The General Regulation provides further detail as to the requirements of the care plan and all nursing and support services.

References to Pain Management, Education in governing or other instruments:

Ontario Reg 79/10 makes reference to pain in respect of the plan of care (s.26), required programs (s.48), pain management (s.52), and training of direct care staff (s.221).

**Manitoba**

Statutes:

Health Services Insurance Act, C.C.S.M, c.H35

Regulations:

Personal Care Homes Standards Regulation, Reg 30/2005

Organization and Administration:

In Manitoba, nursing homes are referred to as a “Personal Care Homes” (PCHs). Manitoba nursing homes must abide by the Personal Care Homes Standards Regulations, under the Health Services Insurance Act. All nursing homes must be licensed. Admissions to long-term care facilities are managed by the Regional Health Authority (RHA) except in Winnipeg, which has a separate Long-Term Care Access Centre within the RHA to manage admissions into long-term care facilities.

Provision of Care

Part 4 of the Personal Care Homes Standards Regulations details the requirements of homes as to resident care, including the initial care plan, integration and review of the plan, incorporation of residents’ wishes and rights to participate in the plan.

References to Pain Management, Education in governing or other instruments:

There is no specific reference to pain in the legislation or regulations.

**Saskatchewan**

Statutes:

Regional Health Services Act, 2002, to be replaced by [The Provincial Health Authority Act.](https://www.ontario.ca/laws/statute/07l08)

Regulations:

The Housing and Special-care Homes Regulations 34/66

The Facility Designation Regulations Ch. R-8.2 Reg 6

Organization and Administration:

In Saskatchewan, the overriding legislation for LTC homes is the Regional Health Services Act. Long-term care homes are designated as “special-care homes” under the Act. The Housing and Special-care Homes Regulations govern the administrative procedures under the Act, including nursing care (section 4), medications (section 8) and food services (section 11). The Facilities Designation Regulations describe the services to be provided by various health-related facilities in the province and section 12 states that a facility designated as a special-care home must provide personal care or nursing care to individuals who reside in the facility.

Provision of Care

The provincial Ministry of Health publishes a manual entitled the Program guidelines for Special-care Homes (the “Guidelines”), last updated in May 2016. Pursuant to the Facility Designation Regulations, section 17(2), all Special-care Homes are required to operate in accordance with the standards set out in the Guidelines. These are considered the minimum standards to be adhered to in publicly-funded facilities that offer LTC services. The Guidelines were written in collaboration with the Regional Health Authorities (RHAs), which are responsible for administration and adherence with the Guidelines at the regional level.

The Guidelines provide detail regarding access to service, types of care to be provided, assessment procedures, requirements and qualifications of nursing and personal care providers, resident care plans, support services and nutrition services.

References to Pain Management, Education in governing or other instruments:

There are no specific references to pain in the statute or regulations. The Care Standards section of the Guidelines, however, stipulate that (u) every effort is made to recognize, assess and appropriately manage pain.

**Alberta**

Statutes:

[Nursing](https://www.ontario.ca/laws/statute/07l08) Homes Act, R.S.A, 2000, Ch. N-7

Regulations:

Nursing Homes General Regulation, Alberta Regulation, 232/1985

Nursing Homes Operation Regulation, Alberta Regulation, 258/1985

Organization and Administration:

In Alberta, LTC homes are governed under the Nursing Homes Act, which defines nursing homes and nursing home care in terms of programs approved by the Minister under the Act. The Act designates the regional health authority as the body that may enter into nursing home contracts with the operators of nursing homes. In addition to governing the terms of contracts, the Act stipulates the terms of operation of nursing homes, including care, payment of benefits, inspections, correction plans, and offenses and penalties.

Provision of Care

The General Regulation defines basic care (section 2) as including accommodation, meals, facilities services, necessary nursing services, personal services, therapeutic and special diets, drugs and medicines, routine dressings and life enrichment services. The Nursing Home Operation Regulation delineates the admission policies of nursing homes, resident assessments, staffing requirements and qualifications. Section 6 of this regulation requires each district board to establish an assessment committee to assess the needs of the person for nursing home care. Section 11 requires that a resident record be maintained and that the charge nurse prepare a description of the resident’s condition, preliminary assessment of capabilities and needs, and a plan for the provision of nursing home care for the resident.

The Nursing Home General Regulation requires that LTC home operators under nursing home contracts be in compliance with two sets of standards set by the Alberta Health Services. The first is the Long-Term Care Accommodation Standards and Checklist, which is set by Alberta Health’s Standards Compliance and Licensing Branch and deals primarily with the physical standards around nursing homes, such as building code, safety, maintenance, personal spaces as well as certain social and nutritional requirements. The second is the Continuing Care Health Service Standards ( CCHSS), set by Alberta Health’s Continuing Care Branch. The CCHSS set the minimum requirement that operators in the continuing care system must meet. Within the residential care sector, the CCHSS apply to both LTC homes and publicly-funded supportive living facilities, and pertain more to person-centered care planning, assessment and case management.

References to Pain Management, Education in governing or other instruments:

The legislation and the regulations do not refer directly to pain assessment or management.

**British Columbia**

Statutes:

Community Care and Assisted Living Act, S.B.C., 2002, Chapter 75.

Regulations:

Residential Care Regulation, B.C. Reg. 96/2009

Organization and Administration:

In British Columbia, the Ministry of Health Services monitors nursing homes and the Ministry of Health Living and Sport establishes legislation, policy, and regulations to protect nursing home residents. Nursing homes in the province can be either publicly or privately owned. All nursing homes in British Columbia that provide three or more prescribed services to three or more persons that are unrelated to them must have a valid license, which must be posted. Nursing homes are required to abide by the Community Care and Assisted Living Act and Regulations.

Provision of Care

Under the definitions provided in the Act, **"care"** includes supervision that is provided to “an adult who is (i) vulnerable because of family circumstances, age, disability, illness or frailty, and (ii) dependent on caregivers for continuing assistance or direction in the form of 3 or more prescribed services.” The Residential Care Regulation, in subsection 2(2), defines the types of care to include “(c) Long-Term Care, being residential care for persons with chronic or progressive conditions, primarily due to the aging process.”

References to Pain Management, Education in governing or other instruments:

None in the legislation or regulations. The Residential Care Regulation includes provisions concerning admission screening, general care requirements and care plans but not specific provisions related to pain or pain management.
